# Zipalertinib—A Novel Treatment Opportunity for Non-Small Cell Lung Cancers with Exon 20 Insertions and Uncommon EGFR Mutations

**DOI:** 10.3390/cancers18020323

**Published:** 2026-01-20

**Authors:** Wolfram C. M. Dempke, Klaus Fenchel, Niels Reinmuth

**Affiliations:** 1 University Hospital, LMU Munich, Grosshadern Campus, 81377 Munich, Germany; 2 Oncology Centre, 07318 Saalfeld, Germany; 3 Asklepios Clinic, Thoracic Oncology, 82131 Gauting, Germany

**Keywords:** NSCLC, targeted therapy, exon 20 insertions, uncommon mutations, zipalertinib

## Abstract

Non-small cell lung cancer (NSCLC) represents over 80% of all lung cancer cases and still has a huge mortality worldwide. Targeting epidermal growth-factor receptor (EGFR) alterations with overall response rates of more than 80% has provided a paradigm shift in the treatment of NSCLC; however, NSCLC patients harbouring uncommon mutations and exon 20 insertions still have a dismal prognosis underscoring the urgent need to develop novel EGFR tyrosine kinase inhibitors (TKIs) with proven activity against these EGFR alterations. Zipalertinib is a newly developed oral, irreversible compound which is characterized by its unique pyrrolopyrimidine structure which discriminates this novel TKI from others. It is active against the classical mutations (i.e., del19, L858R) and some of the uncommon mutations (e.g., T790M, G719X, S768I, L861Q, but not C797S) and is predominantly active in NSCLC cells harbouring exon20ins. Zipalertinib is currently being extensively evaluated in several clinical NSCLC trials (REZILIENT 1–4) and has shown significant clinical activity in NSCLC patients with uncommon mutations, exon20ins, and in brain metastases (REZILIENT 3 trial). Moreover, zipalertinib in combination with platinum-based chemotherapy followed by zipalertinib monotherapy as first-line therapy is currently being evaluated in the pivotal, ongoing REZILIENT 3 randomized trial. In addition, the efficacy of zipalertinib is also studied in the adjuvant setting (REZILIENT 4 trial, stage IB-IIIA NSCLCs with exon20ins and uncommon mutations). The role and the integration of therapies targeting exon20ins or uncommon mutations into the first- and second-line treatment armamentarium for NSCLC patients is not yet fully established, and the therapeutic impact of monotherapies (e.g., sunvozertinib, firmonertinib) versus combinations with standard platinum-based chemotherapy (e.g., zipalertinib, amivantamab) currently still lacks robust evidence to further change the therapeutic landscape for these patients. Therefore, results from the ongoing trials are eagerly awaited and are expected to shed some light on these open questions.

## Simple Summary

Zipalertinib is a novel oral and irreversible targeting uncommon EGFR mutations and exon 20 insertions. The drug is currently undergoing an extensive clinical study programme (REZILIENT 1–4) and results of the ongoing trials will help to better define the role of zipalertinib alone or in combination with chemotherapy for treatment of NSCLC patients.

## 1. Introduction

Non-small cell lung cancer (NSCLC) represents over 80% of all lung cancer cases and still has a huge mortality worldwide. In the Asia-Pacific region, it is also the leading cancer and is one of the major causes of cancer-related deaths [1]. Targeting epidermal growth-factor receptor (EGFR) alterations has provided a paradigm shift in the treatment of NSCLC, and NSCLC harbouring EGFR mutations are detectable in 10–20% of all lung cancer cases in Europe and the USA, and in over 45% of Asian patients with NSCLC [2]. It is generally accepted that alterations in exon 19 (del19) and exon 21 (L858R) represent the most frequent mutations, accounting for approximately up to 90% of EGFR alterations in NSCLC (so-called “classic” or “common” mutations) with generally high sensitivity to tyrosine kinase inhibitor (TKI) treatment.

However, it is important to note that deletions of exon 19 represent a very heterogeneous group of alterations of the intracellular domain of the EGFR gene, with more than 72 variants described in the literature [3,4] with the most frequent one being the E746_A750 (28%) deletion [3]. The vast majority of these alterations is sensitive to approved EGFRmut TKIs; however, the L747-A750>P variant is associated with poor inhibition by erlotinib and osimertinib, but is strongly inhibited by afatinib, which might be due, at least in part, to the structural characteristics of this variant [4].

Most recently, final results from the MARIPOSA-3 trial (osimertinib versus amivantamab plus lazertinib as first-line treatment of NSCLC patients with advanced or metastatic lung tumours harbouring classical mutations) demonstrated a significant benefit for the del19 subgroup in terms of mPFS (18.5 versus 23.9—not estimable) and mOS (HR 0.66) [5,6] suggesting a better activity of the combination than osimertinib in del19 NSCLC patients.

Several lines of evidence from many clinical trials indicated that NSCLC patients with classical mutations have a longer median progression-free survival (mPFS) when treated with TKIs compared with platinum-based chemotherapy alone. Other EGFR mutations are termed “uncommon” (or “atypical”) mutations, and account for up to 18% of all EGFR mutations depending on the detection methodology applied since the polymerase chain reaction (PCR) may detect these alterations but sometimes did not. Therefore, next generation sequencing (NGS) is often needed to more accurately detect rare EGFR mutations and insertions [7].

Uncommon EGFR mutations have been identified to show a variable efficacy to EGFR-targeted drugs depending on the molecular alterations within exons 18–21 [8], which are still not fully clinically evaluated to date, and only afatinib is approved for these mutations so far [9,10], whereas administration of osimertinib, which also shows some activity, is off-label.

**Figure 1:**
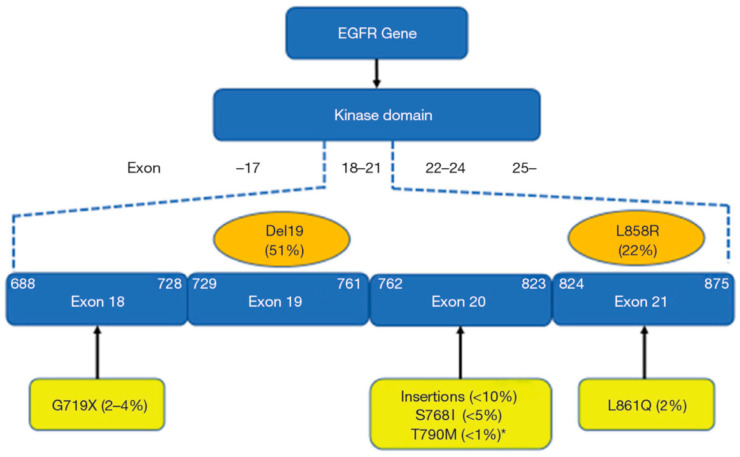
EGFR mutations within exons 18–21 of the tyrosine kinase domain of the gene. Common and uncommon EGFR mutations are depicted by orange and yellow backgrounds, respectively. *: TKI-naïve NSCLC samples [11]. EGFR, epidermal growth-factor receptor; TKI, tyrosine kinase inhibitor; NSCLC, non-small cell lung cancer (adopted from reference [12]).

Amongst the uncommon mutations, the group of exon 20 insertion variants represent a rare and heterogeneous group of EGFR alterations with an incidence of approximately 1–2% of all newly diagnosed NSCLC patients [11] (Figure 1).

Wu et al. [13] provided the first evidence that NSCLC patients harbouring exon 20 insertions had a significantly shorter PFS than those with del19 and L858R mutations (1.4 versus 8.5 months, *p* < 0.001), which adds weight to the proposal that the development of novel drugs targeting uncommon mutations is a high unmet medical need.

The exon 20 insertion mutations are known to be resistant to previously approved EGFR-targeted drugs [14]. However, as demonstrated in several previously published papers and reviews on exon20ins, certain first- and second-generation EGFR-TKIs (e.g., afatinib) also show activity against specific variants (e.g., A763_Y764insFQEA) [15].

Treatment with amivantamab in combination with platinum-based chemotherapy as first-line treatment (PAPILLION trial) [16] and amivantamab (CHRYSALIS trial) [17] or sunvorzertinib (WU-KONG 1B trial) [18] as monotherapy for second-line patients have recently been approved for this patient population.

## 2. Zipalertinib—Preclinical Data

**Figure 2:**
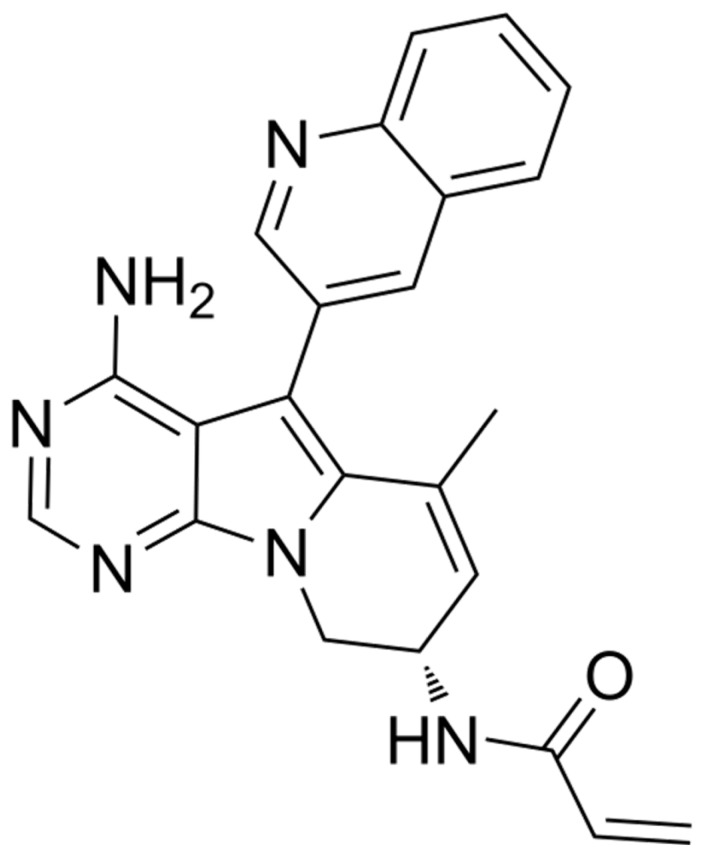
Chemical structure of zipalertinib. Of note, due to the unique pyrrolopyrimidine scaffold, the structure is not related to those of other approved EGFRmut TKIs.

Amongst all EGFRmut TKIs, zipalertinib is a newly developed oral, irreversible compound which is characterized by its unique pyrrolopyrimidine structure (Figure 2) which discriminates this novel TKI from others. These structural differences result in a highly selective, potent, and broad-spectrum efficacy against EGFR mutations [19]. Using NIH/3T3 and Ba/F3 cell lines, IC_50_ values of zipalertinib for EGFR mutations have been reported to be in the range of 1.1 to 8.0 nmol with an IC_50_ ratio (IC_50_ WT/IC_50_mut) of 134, demonstrating that the drug is almost exclusively targeting EGFR mutations [19].

Zipalertinib has been found to have a very pronounced potency and specificity in inhibiting exon 20 insertion mutations within the EGFR gene compared to wild-type EGFR. Experimental tumour models and in vitro systems revealed that zipalertinib binds irreversibly to the cystein797 residue of the intracellular domains of the EGFR which is known to carry the insertions in exon 20 [20].

The amino acid (AA) sequence which is coded by exon 20 spans AAs 762–823 of the EGFR gene (Figure 1), with insertions ranging from short in-frame insertions (three base-pairs: one AA) to duplications with up to 21 base-pairs (7 AAs) [21]. Amongst the over hundred alterations, the vast majority of the exon 20 insertions are rare with 90% of them occurring between AAs 766–775 [21]. The frequent sites are AA 769 (25%), 770 (up to 35%), and 773 (22–26%) [22], with the most common ones being the V769_D770insASV (30%) and D770_N771insSVD (9%).

It should be noted that the different variants show heterogeneity in drug sensitivity which adds weight to the proposal that precise detection of each exon 20 insertion is of importance for clinical treatment decisions. Moreover, in vitro kinase assays have provided further evidence that zipalertinib selectively targets a broad spectrum of exon 20 insertions including D770_N771insNPG [23] while sparing wild-type EGFR, suggesting an enhanced therapeutic window [23].

In addition, further research revealed that zipalertinib is also active against the classical mutations (i.e., del19, L858R) and some of the uncommon mutations (e.g., T790M, G719X, S768I, L861Q), but not against the C797S mutation [19] (Table 1).

## 3. Zipalertinib—Clinical Development Status

### 3.1. REZILIENT 1 Trial

Based on the current preclinical results, the clinical efficacy of zipalertinib was evaluated in a phase I/II open-label trial in NSCLC patients harbouring exon20ins previously being treated with platinum-based chemotherapy (with or without exon20ins-targeted therapies (NCT04036682)). Primary endpoints were overall response rate (ORR) and duration of response (DoR).

A total of 244 patients were treated with zipalertinib (2 × 100 mg daily) (data cut-off December 2024). The primary efficacy population comprised patients who had received prior platinum-based chemotherapy without exon20ins-targeted therapy (125 patients), with amivantamab only (30 patients), or with amivantamab and other exon20ins-targeted therapy (21 patients).

The confirmed ORR was found to be 35.2%, and the median DoR was 8.8 months. In addition, the confirmed ORR was 40%, 30%, and 14.3%, and the median DoR was 8.8, 14.7, and 4.2 months in patients who had received prior platinum-based chemotherapy without exon20ins-targeted therapy, amivantamab only, or amivantamab and other exon20ins-targeted therapy, respectively.

In those 68 patients who presented with brain metastases, the ORR was found to be 30.9%. Reported grade ≥3 treatment-related adverse events comprised anemia (7%), pneumonitis and rash (2.5% each), and diarrhea, ALT increased, and platelet count decreased (2% each). Notably, no grade 3 or higher rash was observed at doses below 150 mg bid [29].

Overall, the reported adverse events were reversible and clinically manageable as reflected in the low rate of dose discontinuation in this trial (8.2%) which adds weight to the observation that zipalertinib combines a good tolerability with an effective target inhibition [29].

The results of this phase I/II suggested that zipalertinib might be an innovative treatment for advanced or metastatic NSCLC patients harbouring exon 20 insertions after prior platinum-based therapy even in those patients with resistance or disease progression following amivantamab therapy. These promising preliminary results prompted the FDA to grant zipalertinib break-through therapy designation [30].

### 3.2. REZILIENT 2 Trial

This is an ongoing multicohort trial (phase IIb, N = 224) evaluating the safety and efficacy of zipalertinib in patients with advanced or metastatic NSCLCs (including those with brain metastases) with EGFR mutations (e.g., exon20ins and uncommon mutations) (Table 2). Cohorts A and B (Table 2) are no longer recruiting patients, and results from cohorts C and D have been most recently published at major congresses: in cohort C (exon20ins and un-common mutations), early results showed an intracranial ORR of 31.3% following zipalertinib monotherapy (all treatment lines) in the evaluable population with measurable disease (N = 16) [31]. The intracranial disease control rate (iDCR) was found to be 68.8% with a median duration of response (DoR) of 8.1 months. Albeit the reported ORR of 31.3% is somewhat lower than those found for sunvozertinib and firmonertinib (52.4% and 46.2%, respectively), one has to take into account that in cohort C heavily pretreated patients have also been enrolled (up to 12 prior treatment lines), whereas only first- and second-line patients received treatment with sunvozertinib or firmonertinib (Table 3). To further clarify the activity in NSCLC patients harbouring exon20ins and brain metastases, the ongoing large phase III trials (zipalertinib: REZILIENT 3; sunvozertinib: WU-KONG28; firmonertinib: FURVENT) will shed some more light on the brain activity of these novel exon20ins TKIs.

In addition, in cohort D (monotherapy in NSCLC patients with uncommon mutation: 50% G719X, 25% L861Q, <25% S768I, N = 40), the confirmed ORR among all patients enrolled was 30% (medium prior lines: 2, range: 1–12). Of note, in the treatment-naïve population (N = 8) the ORR was found to be 62.5%, suggesting a high efficacy of zipalertinib in NSCLC patients harbouring uncommon EGFR mutations [34,35].

### 3.3. REZILIENT 3 Trial

Given the favourable risk–benefit ratio of zipalertinib, its managable safety profile (mainly thrombocytopenias and skin rush), this randomized phase III trial (NCT05973773) investigates zipalertinib in advanced or metastatic first-line NSCLC patients harbouring exon20ins and exon20ins uncommon compound mutations (e.g., exon20ins plus S786I, etc.).

Patients are randomized between platinum (cisplatin or carboplatin) plus pemetrexed chemotherapy and zipalertinib (four cycles) followed by zipalertinib plus pemetrexed versus cisplatin or carboplatin and pemetrexed followed by pemetrexed plus placebo as maintenance therapy. Patients with documented progression in the control arm are eligible for crossover to zipalertinib monotherapy.

Primary endpoint is mPFS; secondary endpoints comprise ORR, mOS, and safety. The trial is currently open for recruitment (N = 266); results are expected in Q3/2026 [34] (Table 3).

### 3.4. REZILIENT 4 Trial

Based on the favourable penetration of the blood-brain-barrier of zipalertinib (demonstrated in the cohort C of the REZILIENT 2 trial) which is critical to prevent early CNS relapses, this randomized phase III trial will evaluate zipalertinib in the adjuvant setting in NSCLC patients (stage IB-IIIA) harbouring exon20ins and/or uncommon mutations (NCT07128199). Patients will be randomized between platinum-based chemotherapy and zipalertinib (after complete tumour resection, four cycles) followed by zipalertinib monotherapy versus platinum-based chemotherapy (after tumour resection, four cycles) followed by placebo.

The primary endpoint will be disease-free survival at three years as assessed by the investigator (N = 360). The trial is not yet open for recruitment; initial results will be expected at the end of 2028 (Table 3).

## 4. Future Directions

In NSCLC patients harbouring exon20ins, CNS involvement still represents a major challenge since effective treatment of brain metastases depends on EGFR TKIs with the potential to cross the blood-brain-barrier (BBB) to achieve high concentrations in the liquor as cellular transporter systems (e.g., P-glycoprotein and others) significantly reduce the TKI accumulation in the brain [36]. In this regard, zipalertinib has shown a good brain penetrance and promising antitumour activity in the REZILIENT 2 trial which will be further investigated in future trials.

Moreover, development of resistance following targeting exon20ins is commonly seen; however, the underlying resistance mechanisms are far from being clear, and non-invasive methods (e.g., liquid biopsies, etc.) to monitor putative resistance mutations are urgently warranted. For instance, in the patients treated with amivantamab, resistance monitoring revealed EGFR amplification and androgen receptor alterations (e.g., H875Y) (amongst several other new mutations) as putative resistance mechanisms [37]. In contrast, C797S and T790M mutations have been identified in mobocertinib-resistant NSCLC patients [25]. These findings require extensive preclinical research to identify strategies to overcome drug resistance in the clinic.

Finally, the role and the integration of therapies targeting exon20ins into the first- and second-line treatment armamentarium for NSCLC patients is not yet fully established, and the therapeutic impact of monotherapies versus combinations with standard chemotherapy currently still lacks robust evidence to further change the therapeutic landscape for these patients. It is generally accepted that the biology and the clinical outcome of NSCLCs with exon20ins is indicative of a highly aggressive tumour phenotype with poor mOS [38,39]. Consequently, both amivantamab and zipalertinib have been combined with platinum-based cytotoxic chemotherapy to attack tumour cells more broadly by causing additional DNA damage. Under the assumption that this strategy may have the potential to eradicate single resistant clones as well as the overgrowth of clones which confer resistance in patients with multiple resistance mutations (e.g., compound mutations), it could thereby contribute to enhance the overall treatment efficacy significantly.

Other strategies, however, have placed emphasis on monotherapy approaches (e.g., firmonertinib and sunvozertinib) to spare patients from the toxic effects of chemotherapy. Albeit initial ORRs were found to be promising in the first- and second-line setting for firmonertinib and sunvozertinib, studies have been conducted mostly in China only. Furthermore, the number of patients enrolled was low, and mPFS results have not yet been reported (Table 4). Therefore, it remains to be seen how durable these results are in the long run and in the light of the unfavourable biology of NSCLCs with exon20ins and exon20ins compound mutations.

## 5. Conclusions

Taken together, the evidence supporting zipalertinib as first-line treatment and in the adjuvant setting still remains unknown; therfore, the mdecial community has not yet established definitive and robust treatment protocols for NSCLC patients harbouring exon20ins and uncommon mutations (neither for monotherapies nor for combinations with platinum-based chemotherapy). As a consequence, clinicians are currently not able to perform adequate assessments between different treatment approaches since we are still lacking sufficient evidence to determine how combination therapies (e.g., zipalertinib or amivantamab with chemotherapy) versus monotherapy (e.g., sunvozertinib or firmonertinib) will affect outcomes for NSCLC patients harbouring exon20ins or uncommon mutations. In addition, the exact mechanisms that cause drug resistance are far from being clear.

Novel small molecules and monoclonal antibodies are currently underway to target the “undruggable” exon 20 insertion in the EGFR gene. Results from ongoing clinical trials are eagerly awaited and will help to further define the role of these compounds in the armamentarium for NSCLC treatment.

## Figures and Tables

**Table 1:** Compounds in late-stage clinical development or FDA/EMA-approved targeting exon 20 insertions in advanced or metastatic non-small cell lung cancer. EGFR, epidermal growth-factor receptor; EMA, European Medical Association; FDA, Food and Drug Administration, USA.

Compound	Targets	Comments (References)
**Poziotinib**	Exon 20 insertions, HER-2/neu	Approved denied by FDA [24]
**Mobocertinib**	Exon 20 insertions, L858R, del18, L861R	FDA approval voluntarily withdrawn (no PFS benefit in the EXCLAIM-2 phase III trial) [25]
**Sunvozertinib**	Exon 20 insertions, L858R, del19, T790M, G719A, L861Q	Second-line: Approval by FDA, phase III ongoing in first-line
**Firmonertinib**	Exon 20 insertions, L858R, del19, T790M, G719X, S768I, L861Q	Approval in China [26], phase II/III trials ongoing
**Silevertinib** **(formerly BDTX-1535)**	Active against almost all common and uncommon mutations; weaker activity against exon 20 insertions	Phase II ongoing [27]
**STX-721**	Exon 20 insertions, L858R, del19, HER2 A775_G776insYVMA (exon 20)	STX-721 demonstrated exon 20 insertion potency and selectivity relative to wild-type (WT) EGFR that surpassed all other tested clinical-phase benchmark EGFR inhibitors suggesting that STX-721 may be less prone to WT EGFR–driven adverse events that have limited the efficacy of other exon 20 insertion inhibitors. Phase II ongoing [28]
**Zipalertinib**	Most EGFR mutation (except C797S), exon 20 insertions	Phase III ongoing
**Amivantamab**	EGFR amplifications, L858R, del19, T790M, G796S, exon 20 insertions, c-MET (monoclonal antibody)	Approved by FDA and EMA

**Table 2:** Development of zipalertinib in clinical trials—the REZILIENT programme. Exon20ins: Exon 20 insertions; PE: primary endpoint; DFS: disease-free survival, ORR: overall response rate; DoR: duration of response.

Trial Name	Design (NCT Number)	Status
**REZILIENT 1**	Phase I/II open-label trial in NSCLC patients harbouring exon20ins previously being treated with platinum-based chemotherapy (with or without exon20ins-targeted therapies) PEs: ORR and DoR (NCT04036682)	Recruitment completed. N = 244 [29]
**REZILIENT 2**	Multicentre cohort trial (phase IIB): Cohort A: prior exon20ins treatment Cohort B: first-line exon20ins treatment Cohort C: active brain metastases (exon20ins, uncommon mutations) Cohort D: uncommon mutations (NCT05967689)	Recruitment ongoing (N = 224 in all cohorts; cohorts A and B closed)
**REZILIENT 3**	Randomized phase III trial in advanced or metastatic first-line NSCLC patients harbouring exon20ins: platinum/pemetrexed chemotherapy and zipalertinib (4 cycles) followed by zipalertinib plus pemetrexed versus platinum/pemetrexed followed by pemetrexed plus placebo maintenance therapy. PE: mPFS (NCT05973773)	Recruitment ongoing (N = 266 planned)
**REZILIENT 4**	Adjuvant randomized phase III trial in NSCLC patients (stage IB-IIIA) harbouring exon20ins and/or uncommon mutations: Platinum-based chemotherapy and zipalertinib (after tumour resection, 4 cycles) followed by zipalertinib monotherapy versus platinum-based chemotherapy (after tumour resection, 4 cycles) followed by placebo. PE: DFS after 3 years. (NCT07128199)	Recruiting ongoing (N = 360 planned)

**Table 3:** Intracranial activity of TKIs targeting exon 20 insertions and uncommon EGFR mutations. The description of the clinical studies listed in this table is largely enumerative since several limitations such as small sample size, geographical specificity, reliance on abstracts data only, and lack of mature mPFS/mOS data impedes an adequate judging of the strength of the evidence. DoR: duration of response, ORR: overall response rate; TL: treatment line; N: number of patients evaluated; PACC: P-loop alpha-C-helix compression.

Drug	N	TL	Mutations	Results	Reference
**Mobocertinib**	40	second-line (after platinum)	Exon20ins	ORR 18% mPFS: 3.7 months	Jänne et al. 2022 [32]
**Firmonertinib**	13	first-line	PACC mutations	ORR (240 mg): 46.2%	Le et al. 2024 [33]
**Sunvozertinib**	21	second-line (after platinum)	Exon20ins	ORR: 52.4%	Yang et al. 2025 [18]
**Zipalertinib**	16	no limit (range: 1–12)	Exon20ins, uncommon mutations	ORR: 31.3% iDCR: 68.8% DoR: 8.1 months	Yu et al. 2025 [31]

**Table 4:** Summary of selected completed and ongoing monotherapy studies with firmonertinib and sunvozertinib in NSCLC patients harbouring exon20ins. 1L: first-line; 2L: second-line, PE: primary endpoint; SE: secondary endpoint; mPFS: median progression-free survival.

Drug	Study	Design	Results	References
**Firmoner-tinib**	FAVOUR (NCT04858958): completed (China only)	Phase Ib Part A: 1L (240 mg, N = 30), Part B: 2L (240 vs. 160 mg, N = 49)	ORR 1L: 78.6% ORR 2L (240 mg): 46.2% ORR 2L (160 mg): 38.5%	Han et al. 2023 [40]
**Firmoner-tinib**	FURVENT (NCT05607550): ongoing (mainly USA and China)	Phase III (1L) Firmonertinib vs. platinum-based chemotherapy (N = 398), PE: mPFS, SE: mOS	Study is active, but not recruiting patients, primary completion expected Q3/2025	www.clinicaltrials.gov (accessed on 4 November 2025)
**Sunvozer-tinib**	WU-KONG 1B (NCT03974022): completed (mainly China and EU)	Phase II (2L) 200 mg (part A) vs. 300 mg (part B)	ORR 46% (2L) ORR 41.7% in amivantamab-pretreated patients FDA approval	Yang et al. 2025 [18]
**Sunvozer-tinib**	WU-KONG 15 (part of WU-KONG 1) (NCT05559645): completed (China only)	Phase II (1L and 2L) N = 28 in 1L (of note: only 1 site in China recruited patients)	ORR 73.1% (1L)	Yang et al. 2023 [41]
**Sunvozer-tinib**	WU-KONG 28 (NCT05668988): ongoing (mainly China and EU)	Phase III (1L) Sunvozertinib vs. platinum-based chemotherapy (N = 320) PE: mPFS, SE: mOS	Study is recruiting patients, completion expected Q1/2026	www.clinicaltrials.gov (accessed on 4 November 2025)

## Data Availability

Data are contained within the article.
